# Ethyl pyruvate reduces liver injury at early phase but impairs regeneration at late phase in acetaminophen overdose

**DOI:** 10.1186/cc11149

**Published:** 2012-01-16

**Authors:** Runkuan Yang, Xiaoping Zou, Marja-Leena Koskinen, Jyrki Tenhunen

**Affiliations:** 1 Department of Critical Care Medicine, University of Pittsburgh Medical School, 3550 Terrace Street, Pittsburgh, PA 15261, USA; 2 Department of Intensive Care Medicine, University of Tampere Medical School, 10 Bio katu, 33521 Tampere, Finland; 3 Department of Gastroenterology, Drum Tower Hospital, Nanjing University Medical School, 321 Zhongshan Street, 210008 Nanjing, China; 4 Department of Pathology, University of Tampere Medical School, 10 Bio katu, 33521 Tampere, Finland

## Abstract

**Introduction:**

Inflammation may critically affect mechanisms of liver injury in acetaminophen (APAP) hepatotoxicity. Kupffer cells (KC) play important roles in inflammation, and KC depletion confers protection at early time points after APAP treatment but can lead to more severe injury at a later time point. It is possible that some inflammatory factors might contribute to liver damage at an early injurious phase but facilitate liver regeneration at a late time point. Therefore, we tested this hypothesis by using ethyl pyruvate (EP), an anti-inflammatory agent, to treat APAP overdose for 24-48 hours.

**Methods:**

C57BL/6 male mice were intraperitoneally injected with a single dose of APAP (350 mg/kg dissolved in 1 mL sterile saline). Following 2 hours of APAP challenge, the mice were given 0.5 mL EP (40 mg/kg) or saline treatment every 8 hours for a total of 24 or 48 hours.

**Results:**

Twenty-four hours after APAP challenge, compared to the saline-treated group, EP treatment significantly lowered serum transaminases (ALT/AST) and reduced liver injury seen in histopathology; however, at the 48-hour time point, compared to the saline therapy, EP therapy impaired hepatocyte regeneration and increased serum AST; this late detrimental effect was associated with reduced serum TNF-α concentration and decreased expression of cell cycle protein cyclin D1, two important factors in liver regeneration.

**Conclusions:**

Inflammation likely contributes to liver damage at an early injurious phase but improves hepatocyte regeneration at a late time point, and prolonged anti-inflammation therapy at a late phase is not beneficial.

## Introduction

Acetaminophen (APAP) hepatotoxicity is the major cause of acute liver failure in the US and Europe [1]. Massive necrosis of the hepatocyte is the feature of APAP-induced acute liver injury (ALI) [2,3]. Currently, the underlying mechanisms of APAP toxicity are still not clear, but inflammation is certainly involved in the pathogenesis of APAP hepatotoxicity. Neutrophils and lipopolysaccharide (LPS) have been shown to contribute to liver injury in APAP overdose [4,5], and pro-inflammatory mediator tumor necrosis factor-alpha (TNF-α) has been shown to promote tissue damage [6-8]. However, conflicting data are also reported: a significant reduction in neutrophil accumulation shows no protection against APAP toxicity [9], and TNF-α is shown to have a role in repair in APAP liver injury [10]. It is also reported that Kupffer cell depletion confers protection at early time points after APAP treatment [11] but can lead to a more severe injury at a later time point [12]. These data suggest that there is a possibility that some inflammatory factors contribute to APAP liver injury at an early injurious phase but that these factors might also facilitate liver regeneration at a late phase.

Tissue repair is an important determinant of final outcome of toxicant-induced injury [13,14]. Since hepatocytes are mostly in a quiescent state (G_0_) [15], pro-inflammatory cytokines such as TNF-α and interleukin-6 (IL-6) [15-17] are needed to prime hepatocytes. This process makes cells more responsive to growth factors. The exposure to hepatocyte growth factor results in the expression of cell cycle proteins [15]. The induction of cyclin D1 is the most reliable marker for cell cycle (G_1 _phase) progression in hepatocytes [15]. Once hepatocytes express cyclin D1, they have passed the G_1 _restriction point and are committed to DNA replication [15].

Ethyl pyruvate (EP) is a potent anti-inflammatory agent and reactive oxygen species (ROS) scavenger [18,19]. EP can inhibit LPS-stimulated macrophages to release TNF-α and IL-6 [20]. EP also protects against liver injury in a couple of murine models such as acute alcoholic hepatitis [21], hemorrhagic shock [22], sepsis [23], acute extrahepatic obstruction [24], and acute necrotizing pancreatitis [25].

In light of this information, we hypothesize that inflammation might contribute to liver injury at an early injurious phase but facilitate liver regeneration at a late time point. We tested this hypothesis by using EP, a potent anti-inflammatory agent, to treat APAP overdose for 24 or 48 hours in a murine model.

## Material and methods

### Materials

All chemicals were purchased from Sigma-Aldrich (St. Louis, MO, USA) unless noted otherwise.

### Animal model and experimental groups

This research protocol complied with regulations published by the National Institutes of Health in regard to the care and use of experimental animals and was approved by the Institutional Animal Use and Care Committee of the University of Pittsburgh Medical School. Male C57BL/6 mice weighing 20 to 25 g (The Jackson Laboratory, Bar Harbor, ME, USA) were used in this study. The animals were maintained at the University of Pittsburgh Animal Research Center with a 12-hour light-dark cycle and free access to standard laboratory food and water. The animals were fasted overnight prior to the experiments.

A single dose (40 mg/kg) of EP has been shown to be consistently effective to protect against liver injury in the following animal models: acute alcoholic hepatitis [21], hemorrhagic shock [22], sepsis [23], acute extrahepatic obstruction [24], and acute necrotizing pancreatitis [25]. Therefore, 40 mg/kg of EP was chosen to treat APAP overdose in this study. The pilot experiments showed that serum alanine aminotransferase/aspartate aminotransferase (ALT/AST) began to rise 2 hours after APAP administration. Therefore, we started the treatments 2 hours after APAP injection, when the liver injury began to occur.

In the first experiment, ALI was induced by a single dose of APAP (350 mg/kg dissolved in 1 mL of sterile saline) administered by intraperitoneal (i.p.) injection. Fourteen APAP-challenged mice were then randomly assigned to the EP group (*n *= 7) or the saline group (*n *= 7). Six mice injected with saline not containing APAP served as the control group. Two hours after APAP injection, the animals in the EP group were intraperitoneally injected with 0.5 mL of EP solution (40 mg/kg dissolved in 0.5 mL of sterile saline) every 8 hours. The same amount of saline was given to the saline group or the control group at equivalent time points. Twenty-four hours after APAP injection, all surviving mice in each group were anesthetized with sodium pentobarbital (90 mg/kg i.p.), serum was collected to measure AST and ALT, and the left lobe of the liver was stored in 10% formalin for pathology (hematoxylin-eosin, or HE, staining).

In the second experiment, three separate groups of mice were used. ALI model and treatment remained the same as in the first experiment except the treatment was extended to 48 hours. Forty-eight hours after APAP injection, all surviving mice in each group were anesthetized with sodium pentobarbital (90 mg/kg i.p.), and the following procedures were performed: blood was aspirated from the heart to measure serum ALT/AST, the left lobe of the liver was stored in 10% formalin for pathology (HE staining), and the rest of the liver tissue was harvested and frozen for protein extractions.

In the third experiment, three separate groups of mice were used. ALI model and treatment remained the same as in the second experiment except the treatment was given for the first 24 hours only; no further treatment was given for the 24- to 48-hour time point. Forty-eight hours after APAP injection, all surviving mice in each group were anesthetized with sodium pentobarbital (90 mg/kg i.p.), serum was collected to measure ALT/AST, and the left lobe of the liver was stored in 10% formalin for pathology (HE staining).

In the fourth experiment, three separate groups of mice were used. ALI model and treatment remained the same as in the second experiment except the treatment was given for the 24- to 48-hour time point only; no treatment was given for the first 24 hours. Forty-eight hours after APAP administration, all surviving mice in each group were anesthetized with sodium pentobarbital (90 mg/kg i.p.), serum was collected to measure ALT/AST, and the left lobe of the liver was stored in 10% formalin for pathology (HE staining).

### Serum aminotransferase measurements

Serum levels of AST and ALT were measured at 37°C with a commercially available kit (from Sigma Diagnostic, part of Sigma-Aldrich).

### Histological analysis

Consecutive sections (5 μm) from paraffin-embedded liver were prepared for HE staining. The percentage of necrosis was estimated by evaluating the number of microscopic fields with necrosis in comparison with the entire cross-section. In general, necrosis was estimated at low power (100 ×) and questionable areas were evaluated at higher magnification (200 ×). The pathologist evaluated all histological sections in a blinded fashion. Inflammatory cell infiltration results were scored semi-quantitatively by averaging the number of inflammatory cells per microscopic field at a magnification of 200 ×. Five fields per tissue sample were evaluated, and six animals in each group were examined.

### Serum TNF-α and IL-6 concentrations

Blood was obtained by cardiac puncture, and the serum was collected and stored frozen at -80°C until assayed for IL-6 and TNF-α by using enzyme-linked immunosorbent assay kits from R&D Systems Inc. (Minneapolis, MN, USA) in accordance with the instructions of the manufacturer.

### Tissue myeloperoxidase

Neutrophil infiltration was measured at 24 and 48 hours by determining myeloperoxidase (MPO) activity in liver tissue homogenates as previously described [26] and was used as an index of neutrophil infiltration in all groups. The MPO levels were expressed as units per gram of tissue (U/g).

### Hepatic tissue malondialdehyde

This assay for lipid peroxidation was performed at 24 and 48 hours as previously described [21]. Results were expressed as nanomoles of malondialdehyde (MDA) per gram of tissue.

### Western blot

Liver protein was extracted as previously described [27]. Equivalent amounts of protein were boiled in sample buffer and separated on 7.5% pre-cast SDS-polyacrylamide gels (Bio-Rad Laboratories, Inc., Hercules, CA, USA) and transferred to nylon membranes. Membranes were then probed with a specific antibody against cyclin D1 (Cell Signaling Technology, Inc., Danvers, MA, USA) protein, visualized with an enhanced chemiluminescence substrate (Amersham Pharmacia Biotech, now part of GE Healthcare, Little Chalfont, Buckinghamshire, UK), and exposed to x-ray film in accordance with the instructions of the manufacturer.

### Hepatocyte proliferation (DNA synthesis)

At the 24-hour time point, the APAP-challenged mice showed only occasional hepatocyte nuclei labeled for 5-bromo-2-deoxyuridine (BrdU) [28]. Therefore, the BrdU test was performed at only the 48-hour time point in this study. To evaluate hepatocyte regeneration, mice from the 48-hour groups were administered BrdU (50-mg/kg i.p. injection at the 46-hour time point) 2 hours before they were killed. Parraffin-embedded liver sections were prepared and processed for immunohistochemistry by using BrdU *in situ *staining kits from BD Pharmingen (San Jose, CA, USA) in accordance with the instructions of the manufacturer. Digital images of five low-power fields from each liver were obtained in a random and blinded fashion, and the number of BrdU-labeled hepatocyte nuclei was counted. The average number of BrdU-positive hepatocytes in each animal was used for subsequent analysis.

### Statistical methods

Results are presented as mean ± standard error of the mean (SEM). Continuous data were analyzed by using the Student *t *test or analysis of variance followed by the Fisher least significant difference test. *P *values of less than 0.05 were considered significant. Summary statistics are presented for densitometry results from studies using Western blot for cyclin D1 expression, but these results were not subjected to statistical analysis since the method employed was only semi-quantitative (*n *= 6).

## Results

### Serum alanine aminotransferase/aspartate aminotransferase

**Figure 1:**
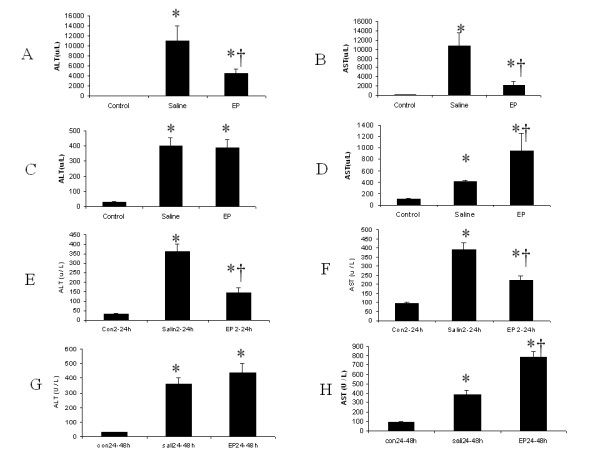
**Effect of treatment with saline or ethyl pyruvate on serum transaminases (ALT/AST) in acetaminophen (APAP)-induced acute liver injury (ALI) model**. **(A, B) **ALI was induced in C57Bl/6 male mice with a single dose of APAP (350 mg/kg) by intraperitoneal (i.p.) injection. Two hours after APAP injection, the animals were treated with 0.5 mL of saline or 0.5 mL of ethyl pyruvate (40 mg/kg) every 8 hours. ALT and AST were measured 24 hours after APAP injection (*n *= 6 survival mice for each group). **(C, D) **Three separate groups of mice were used. ALI model and treatments were the same as in (A) and (B) except the treatment was extended to 48 hours. ALT and AST were measured at 48 hours after APAP injection (*n *= 6 survival mice for each group). **(E, F) **Three separate groups of mice were used. ALI model and treatments were the same as in (C) and (D) except there was no further treatment for the 24- to 48-hour time point. ALT and AST were measured at 48 hours after APAP injection (*n *= 6 survival mice for each group). **(G, H) **Three separate groups of mice were used. ALI model and treatments were the same as in (C) and (D) except the treatment was given only for the 24-hour to 48-hour time point. ALT and AST were measured at 48 hours after APAP injection (*n *= 6 survival mice for each group). Results are presented as mean ± standard error of the mean. *P < 0.05 versus control; †*P *< 0.05 versus saline. ALT, alanine aminotransferase; AST, aspartate aminotransferase.

Twenty-four hours after APAP injection, one mouse from the saline group and one mouse from the EP group died, and all mice in the control group survived. Compared with saline treatment, EP therapy decreased serum concentrations of ALT/AST by a statistically significant degree (Figure 1A, B). However, 48 hours after APAP challenge, EP-treated mice showed a significantly higher serum AST concentration in comparison with the saline treatment group (Figure 1C, D). Compared with early-phase (2 to 24 hours) saline treatment, early-stage (2 to 24 hours) EP treatment significantly decreased serum ALT/AST concentrations at 48 hours (Figure 1E, F). However, compared with late-phase (24- to 48-hour) saline treatment, late-stage (24- to 48-hour) EP administration significantly increased serum AST concentration (*P < 0.05 versus the control group, †*P *< 0.05 versus the saline group, *n *= 6 for each group) (Figure 1G, H).

### Liver histopathology

**Figure 2:**
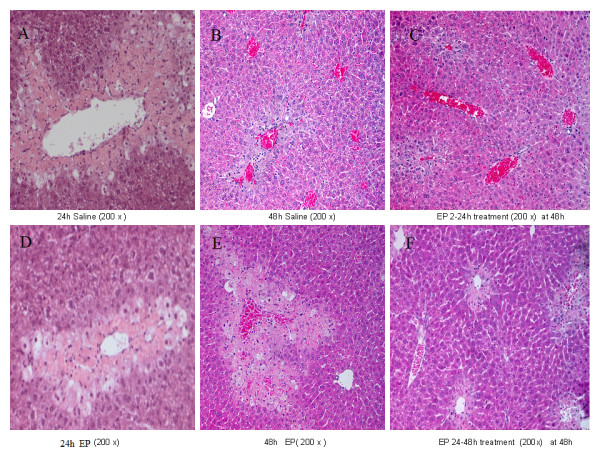
**Effect of treatment with saline or ethyl pyruvate (EP) on pathology in acetaminophen-challenged mice**. Hematoxylin-eosin staining was assessed 24 and 48 hours after induction of acute liver injury (or sham procedure). Method and treatment were the same as described in Figure 1 (*n *= 6 for each group). Since the three saline-treated groups (saline 2- to 48-hour group, saline 2- to 24-hour group, and saline 24- to 48-hour group) showed comparable pathology, only saline 2- to 48-hour treatment at 48 hours is shown. A typical picture is shown. (A = 24 h Saline, B = 48 h Saline, C = 2-24 h EP, D = 24 h EP, E = 48 h EP, F = 24-48 h EP).

Twenty-four hours after APAP injection, the saline-treated mice showed 58.4% ± 7.3% necrotic area and a large number of infiltrating inflammatory cells (386 ± 28 per high-power field, *n *= 6); EP treatment statistically reduced the necrotic area to 35.6% ± 4.6% and significantly decreased the number of infiltrating inflammatory cells (261 ± 17 per high-power field, *n *= 6, *P *< 0.05). Cell boundary loss and ballooning degeneration were also found around the hepatic central vein in the saline and EP groups. In histological evaluation 48 hours after ALI induction, the saline-treated mice demonstrated 8.6% ± 1.5% necrotic area, evident regeneration, and extensive infiltration of inflammatory cells (242 ± 35 per high-power field, *n *= 6) in the centrilobular regions. In contrast, EP-treated mice showed statistically larger necrosis (26.6% ± 3.3%) in the centrilobular region and no evident regeneration was seen in the EP group; treatment with EP statistically reduced the number of infiltrating inflammatory cells (156 ± 24 per high-power field, *n *= 6). Forty-eight hours after APAP administration, the early-phase (2- to 24-hour) saline-treated mice and the late-phase (24 to 48 hours) saline-treated animals showed hepatic necrosis and inflammatory cell infiltration comparable to those of the 48-hour saline group (2 to 48 hours) as described above. Compared with saline treatment, early EP treatment statistically decreased hepatic necrosis (3.4% ± 0.3%, *P *< 0.05) and reduced the number of infiltrating inflammatory cells (160 ± 28 per high power field, *n *= 6) at 48 hours. In contrast, the late EP administration group showed significantly increased necrosis (23.2% ± 4.2%, *P *< 0.05, *n *= 6) and reduced the number of infiltrating inflammatory cells (150 ± 34 per high-power field, *n *= 6) in comparison with the 48-hour saline group. The hepatic necrosis in the EP (24- to 48-hour) group was not statistically smaller than that in the EP (2- to 48-hour) group at 48 hours (*P *> 0.05). Since the three saline-treated groups (saline 2- to 48-hour group, saline 2- to 24-hour group, and saline 24- to 48-hour group) at 48 hours showed comparable pathology, only saline 2- to 48-hour treatment at 48 hours is shown in Figure 2. Necrosis data are presented in Table 1.

**Table 1:** Hepatic necrosis at 24 and 48 hours after acetaminophen administration

Animal groups	24 hours	48 hours
Saline (2 to 48 hours)	58.4 ± 7.3	8.6 ± 1.5
Ethyl pyruvate (2 to 48 hours)	35.6 ± 4.6^a^	26.6 ± 3.3^a^
Saline (2 to 24 hours)		8.8 ± 1.5
Ethyl pyruvate (2 to 24 hours)		3.4 ± 0.3^a^
Saline (24 to 48 hours)		8.3 ± 1.2
Ethyl pyruvate (24 to 48 hours)		23.2 ± 4.2^a^

Hepatic necrosis is expressed as a percentage. Acetaminophen dose was 350 mg/kg. Six mice were in each group. ^a^*P *< 0.05 between the saline treatment and the ethyl pyruvate therapy.

### Serum TNF-α and IL-6 concentrations

**Figure 3:**
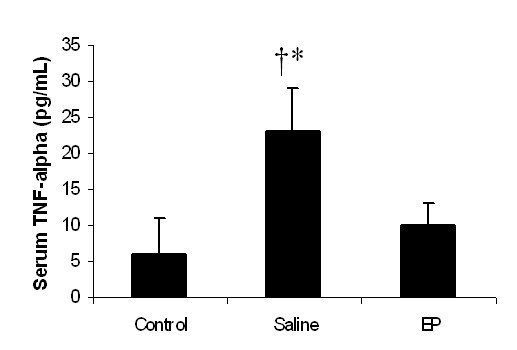
**Effect of treatment with saline or ethyl pyruvate (EP) on serum tumor necrosis factor-alpha (TNF-α) in acetaminophen-treated mice**. Serum TNF-α was assessed at 48 hours after the induction of acute liver injury (or sham procedure). Results are presented as mean ± standard error of the mean (*n *= 6). *P < 0.05 versus control; †*P *< 0.05 versus EP.

Twenty-four hours after APAP injection, the serum TNF-α concentration in the control group was 5.1 ± 3.4 pg/mL, and the TNF-α concentrations in the saline and EP groups remained the same as in the control group (*P *> 0.05, *n *= 6 for each group). However, at 48 hours, the serum TNF-α concentration in the saline group was statistically higher than those in the control and EP groups (*P *< 0.05, *n *= 6 in each group) (Figure 3), and there was no statistically significant difference between the control and EP groups. Serum IL-6 concentration was undetectable in each group at both the 24-hour and the 48-hour time points.

### Hepatic tissue myeloperoxidase levels

**Figure 4:**
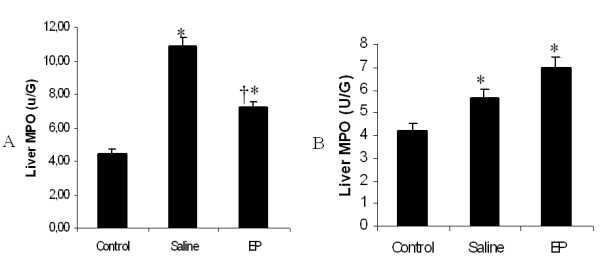
**Effect of treatment with saline or ethyl pyruvate (EP) on hepatic myeloperoxidase (MPO) activity in mice with acute liver injury (ALI)**. Liver MPO was assessed 24 hours **(A) **and 48 hours **(B) **after induction of ALI (or sham procedure). Results are presented as mean ± standard error of the mean (*n *= 6). *P < 0.05 versus control; †*P *< 0.05 versus EP.

Tissue MPO activity was determined as an index of neutrophil infiltration after APAP injection in the liver. At the 24-hour time point, the mean liver MPO activity value for the control group was 4.3 ± 0.3 U/g, and this value significantly increased to 10.8 ± 0.4 U/g in the saline group and 7.1 ± 0.3 U/g in the EP group (Figure 4A) (*P < 0.05). Compared with saline treatment, EP treatment significantly reduced MPO values (*P *< 0.05). Forty-eight hours after APAP administration, the mean liver MPO value for the control group was 4.3 ± 0.3 U/g, and this value significantly increased to 6.0 ± 0.3 U/g in the saline group and 6.9 ± 0.4 U/g in the EP group (Figure 4B) (*P < 0.05), but there was no statistically significant difference between the saline group and the EP group (*n *= 6 for each group, data were shown as mean ± SEM, *P < 0.05 versus the control group, †*P *< 0.05 versus the saline group).

### Hepatic tissue malondialdehyde concentrations

Neither 24-hour nor 48-hour hepatic tissue MDA concentrations were significantly changed in the saline or EP groups in comparison with the normal control group (*P *> 0.05) (Table 2).

**Table 2:** Effect of treatment with saline or ethyl pyruvate on liver malondialdehyde concentrations in the control or acetaminophen-injected mice

Animal groups	24 hours	48 hours
Control	8.1 ± 1.4	8.0 ± 1.3
Saline	9.3 ± 1.6	9.1 ± 1.4
Ethyl pyruvate	8.3 ± 1.3	8.3 ± 1.2

Malondialdehyde concentrations are expressed as nanomoles per gram of tissue and presented as mean ± standard error of the mean. Acetaminophen dose was 350 mg/kg. Six mice were in each group. *P *> 0.05 among each groups.

### Hepatic cyclin D1 expression

**Figure 5:**
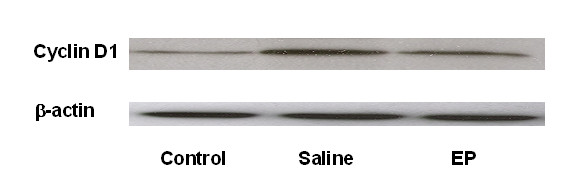
**Effect of treatment with saline or ethyl pyruvate on cyclin D1 expression in the hepatic tissue**. Western blot was performed by using hepatic extracts prepared from tissues obtained 48 hours after acetaminophen injection. Results from six representative assays are shown (*n *= 6). Typical gels are depicted. EP, ethyl pyruvate.

The timely onset of tissue repair processes can limit liver injury and promote regeneration of lost tissue mass [14]. The induction of cyclin D1 is the most reliable marker for cell cycle (G_1 _phase) progression in hepatocytes [15]. Western blot was performed by using whole-cell extracts prepared from liver tissue to assess expression of cyclin D1 in mice subjected to ALI or the control procedure. In Figure 5, cyclin D1 expression in the control group and EP group was minimal. In contrast, cyclin D1 expression was clearly observed in the saline-treated animals at 48 hours after APAP administration.

### Hepatic BrdU expression

**Figure 6:**
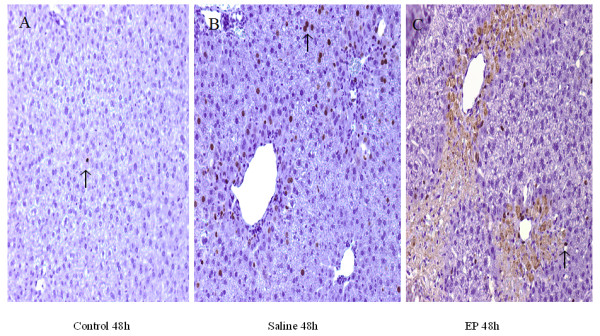
**Effect of treatment with saline or ethyl pyruvate (EP) on hepatocyte regeneration in acetaminophen-injected mice**. BrdU (5-bromo-2-deoxyuridine) staining was assessed at 48 hours after induction of acute liver injury (or sham procedure, *n *= 6 for each group). A typical picture is shown. BrdU-positive nuclei are indicated by arrows.(A = 48 h Control, B = 48 h Saline, C = 48 h EP).

The hepatocyte proliferation was assessed by BrdU immunohistological staining. BrdU-positive nuclei are shown by the arrows. At 48 hours, the number of labeled nuclei (per low power) was significantly increased in both saline (74 ± 10) (Figure 6B) and EP (20 ± 5) (Figure 6C) groups though to a statistically lesser extent in the EP-treated mice. In addition, the EP group showed a brown background staining in the cell plasma of the large necrotic area.

## Discussion

The purpose of this study was to test the hypothesis that inflammation might contribute to liver damage at an early injurious phase but facilitate liver regeneration at a late phase in APAP overdose. The major novel findings of this investigation are the following: (a) EP-treated mice demonstrate decreased serum ALT/AST and reduced necrosis at 24 hours; however, EP therapy shows increased serum AST and impaired liver regeneration at 48 hours; (b) the late detrimental effect is associated with a decreased serum TNF-α concentration; and (c) EP-treated mice demonstrate significantly reduced expression of cell cycle protein cyclin D1 in liver tissue.

EP-treated mice demonstrated decreased serum ALT/AST and reduced necrosis at 24 hours. This early protective effect was associated with significantly decreased hepatic MPO and a reduced number of infiltrating inflammatory cells in comparison with the saline-treated group. In this study, we also measured 24-hour and 48-hour hepatic MDA levels, a parameter of hepatic lipid peroxidation, which were not significantly changed in the saline or EP groups in comparison with the normal control group. This MDA result did not support the notion that the beneficial effect of EP in the early phase could be attributed to the antioxidant effects of EP. The serum TNF-α and IL-6 concentrations were not elevated at 24 hours in this model. This result was unexpected, and it is possible that 24 hours was not the peak time for serum TNF-α and IL-6 in this model.

EP impaired hepatocyte regeneration at 48 hours, and this result was unexpected. Therefore, we repeated the experiment once more with another dose (80 mg/kg) of EP, and the result at the 48-hour time point was comparable to that of the 40-mg/kg dose. The result did not show a clear dose-dependent pattern in this model. Therefore, this study focused on the 40-mg/kg dose of EP. Currently, there is no evidence that continued therapy with EP might have prevented late-phase toxicity of APAP. If EP prevented late-phase toxicity of APAP, EP treatment would be beneficial at 48 hours. However, in this study, compared with the saline therapy, the EP (2 to 48 hours) treatment and the late-phase EP (24 to 48 hours) therapy both showed increased hepatic necrosis at the 48-hour time point.

Massive hepatocyte necrosis is the predominant feature of APAP-induced ALI. Liver regeneration is vital for survival after a toxic insult [14], and cyclin D1 is an important cell cycle protein [15]. In this study, Western blot showed that EP treatment markedly decreased the level of cyclin D1 in the APAP-challenged liver tissue. The decreased cyclin D1 expression was associated with increased serum AST and impaired liver regeneration in EP-treated mice receiving APAP, suggesting that EP therapy likely impairs liver regeneration via a cyclin D1-mediated pathway.

EP-treated mice demonstrated higher serum AST and impaired hepatocyte regeneration at the 48-hour time point. This could be due to the decreased pro-regenerative cytokine TNF-α level because TNF-α might prime hepatocytes for regeneration.

After 24 hours, MPO levels and the number of inflammatory cells were lower in the EP group in comparison with the saline group. This could be secondary to the significantly larger degree of necrosis seen in the saline group after 24 hours. In this investigation, at the 48-hour time point, EP treatment did not reduce MPO level in comparison with the saline therapy. This is probably because EP delayed hepatocyte regeneration and a larger necrotic area remained in the EP group, and the large necrotic tissue might attract more neutrophil infiltration.

In this study, compared with the 48-hour saline group, early-phase (2 to 24 hours) EP-treated mice demonstrated statistically smaller necrosis at 48 hours. It is likely that early EP treatment reduced liver injury at 24 hours. Therefore, the EP group might have smaller hepatic necrosis at the 24-hour time point in comparison with the saline group. Smaller necrosis in the EP group might heal more quickly than the bigger necrosis in the saline group while approaching the 48-hour time point, and early EP therapy probably did not markedly impair hepatocyte regeneration. In contrast, late-phase (24 to 48 hours) EP therapy (without early-phase treatment) showed significantly increased hepatic necrosis at 48 hours in comparison with the saline group, suggesting that inflammation facilitates hepatocyte regeneration at a late phase, and anti-inflammatory therapy at a late phase is not beneficial.

Currently, N-acetyl-cysteine (NAC), a glutathione precursor, is the antidote for APAP overdose [29]. However, this antidotal therapy is effective for early-presenting patients and is less effective for late-presenting patients [29,30]. Therefore, additional therapies are needed. In this study, EP therapy protected against liver injury at an early phase. However, at a late time point, even though there was a large number of infiltrating inflammatory cells, the anti-inflammation therapy with EP impaired hepatocyte regeneration. More investigations are needed to reevaluate the role of inflammation in APAP hepatotoxicity.

## Conclusions

Inflammation likely contributes to liver injury at an early phase but facilitates hepatocyte regeneration at a late time point in APAP hepatotoxicity, and prolonged anti-inflammation therapy at a late phase is not beneficial.

## Key messages

• Ethyl pyruvate (EP) treatment reduces liver injury at 24 hours after acetaminophen (APAP) challenge. This early protective effect is associated with decreased hepatic myeloperoxidase and a reduced number of infiltrating inflammatory cells.

• EP therapy impairs liver regeneration at 48 hours after APAP overdose. This effect is associated with reduced serum tumor necrosis factor-alpha and decreased cyclin D1 level.

## Abbreviations

ALI: acute liver injury; ALT: alanine aminotransferase; APAP: acetaminophen; AST: aspartate aminotransferase; BrdU: 5-bromo-2-deoxyuridine; EP: ethyl pyruvate; HE: hematoxylin-eosin; IL-6: interleukin-6; i.p.: intraperitoneal; LPS: lipopolysaccharide; MDA: malondialdehyde; MPO: myeloperoxidase; SEM: standard error of the mean; TNF-α: tumor necrosis factor-alpha.

## Competing interests

The authors declare that they have no competing interests.

## Authors' contributions

RY designed the study. All authors participated in the animal handling and procedures and read and approved the final manuscript.
